# An In Silico Study for Expanding the Utility of Cannabidiol in Alzheimer’s Disease Therapeutic Development

**DOI:** 10.3390/ijms242116013

**Published:** 2023-11-06

**Authors:** Kyudam Choi, Yurim Lee, Cheongwon Kim

**Affiliations:** 1 Heerae Co., Ltd., Seoul 06253, Republic of Korea; kch38896@naver.com; 2 Department of Software, Sejong University, Seoul 05006, Republic of Korea; yurimlee714@gmail.com

**Keywords:** cannabidiol, Alzheimer’s disease, target identification, drug optimization, molecular docking, molecular dynamics

## Abstract

Cannabidiol (CBD), a major non-psychoactive component of the cannabis plant, has shown therapeutic potential in Alzheimer’s disease (AD). In this study, we identified potential CBD targets associated with AD using a drug-target binding affinity prediction model and generated CBD analogs using a genetic algorithm combined with a molecular docking system. As a result, we identified six targets associated with AD: Endothelial NOS (ENOS), Myeloperoxidase (MPO), Apolipoprotein E (APOE), Amyloid-beta precursor protein (APP), Disintegrin and metalloproteinase domain-containing protein 10 (ADAM10), and Presenilin-1 (PSEN1). Furthermore, we generated CBD analogs for each target that optimize for all desired drug-likeness properties and physicochemical property filters, resulting in improved pIC50 values and docking scores compared to CBD. Molecular dynamics (MD) simulations were applied to analyze each target’s CBD and highest-scoring CBD analogs. The MD simulations revealed that the complexes of ENOS, MPO, and ADAM10 with CBD exhibited high conformational stability, and the APP and PSEN1 complexes with CBD analogs demonstrated even higher conformational stability and lower interaction energy compared to APP and PSEN1 complexes with CBD. These findings demonstrated the capable binding of the six identified targets with CBD and the enhanced binding stability achieved with the developed CBD analogs for each target.

## 1. Introduction

Cannabis, which contains more than 700 chemical compounds, has traditionally been used in various industries, including food, pharmaceuticals, and textiles. Two major compounds in cannabis are tetrahydrocannabinol (THC) and cannabidiol (CBD), which are known as cannabinoids [1]. THC is a major psychoactive component of the cannabis plant and is classified as a narcotic. On the contrary, CBD, a major non-psychoactive component of the cannabis plant, has polypharmacological properties and has been extensively evaluated for various disease indications. Clinical studies on CBD have shown its efficacy in treating multiple clinical conditions, such as chronic pain, depression, anxiety disorders, sleep disorders, and psychosis [2,3]. Additionally, CBD has been recognized as a typical therapeutic and preventive agent for neurological diseases, including anxiety, depression, and epilepsy [4,5,6,7], and neurodegenerative diseases, including Parkinson’s disease, Alzheimer’s disease (AD), and amyotrophic lateral sclerosis [5,8,9].

AD is one of the most common neurodegenerative diseases, with no treatment or prevention measures but only limited symptom relief [10]. AD majorly has two biomarkers, which are senile plaques made of β-amyloid protein and hyperphosphorylated tau-induced neurofibrillary tangles [10,11]. Recently, several works have indicated CBD’s role in treating and preventing AD. CBD has been shown to reduce tau protein hyperphosphorylation [12] and accumulation of β-amyloid protein [13] and to have anti-inflammatory and antioxidant properties [14,15,16].

Previous works have been introduced in two approaches to expand CBD’s role in treating and preventing AD. The first approach was identifying targets associated with AD by which CBD was activated. Especially, several works have shown that CBD has a low affinity for classic targets, such as cannabinoid receptor type 1, 2 (CB1, CB2), despite acting as an antagonist/inverse agonist in them [17,18,19,20], so they identify potential targets, such as non-endocannabinoid G protein-coupled receptor 3, 55, 6 (GPR3, GPR55, GPR6), transient receptor potential vanilloid type 1 (TRPV1), and peroxisome proliferator-activated receptors (PPARs), and indicate the pharmacological role of CBD on targets in vitro, and in vivo [13,21,22,23,24]. The second approach was generating CBD analogs that increase affinities on targets, have a wide range of anti-inflammatory activities, and optimize their properties [25,26,27]. CBD analogs were generated experimentally, and most of their biological properties and activities are indicated in vitro. Therefore, existing CBD targets and analogs have demonstrated their utility, but their generation steps are relatively time-consuming and costly compared in silico.

In this paper, we identified six potential CBD targets associated with AD and generated CBD analogs that can interact with each target through in in silico methods. Subsequently, we explored the potential of these CBD targets and analogs using extensive analyses. Potential targets were identified using our target identification method, which consisted of molecular similarity and a drug–target binding affinity prediction model called DeepPurpose [28]. These targets demonstrated interaction with CBD based on drug–target binding affinity and molecular docking. To generate CBD analogs, we employed our molecular-constrained optimization method [29], which combined a genetic algorithm (GA)-based method with molecular docking. This method automatically modified the molecular substructure while maintaining the scaffold, aiming to achieve a lower docking score to the protein target and optimize desired drug-likeness properties and physicochemical filters. Finally, the structural stability of CBD-identified target complexes and CBD analog-identified target complexes were analyzed through molecular dynamics (MD) simulations.

## 2. Results

### 2.1. Identified Potential CBD Target Related to AD

We extracted 74 CBD-like molecules with an average fingerprint Tanimoto similarity score higher than 0.4 between CBD and other molecules. Subsequently, we calculated the binding affinity between each CBD and the CBD-like molecules’ targets using DeepPurpose. The binding affinity was calculated via the log scale of the half-maximal inhibitory concentration ($pIC_{50}$ value). The $pIC_{50}$ value depended on the concentrations of the target and drug molecules, where an increased $pIC_{50}$ value indicated a stronger binding affinity. We initially selected 3646 potential targets having higher than 4.0 $pIC_{50}$ values in both CBD and CBD-like molecules and further filtered targets associated with AD among potential targets. As a result, we identified six related to AD as potential targets: Endothelial NOS (ENOS), Myeloperoxidase (MPO), Apolipoprotein E (APOE), Amyloid-beta precursor protein (APP), Disintegrin and metalloproteinase domain-containing protein 10 (ADAM10), and Presenilin-1 (PSEN1). This result is shown in Table 1. The $pIC_{50}$ values of these potential targets ranged from 4.81 to 7. The $pIC_{50}$ values of known targets predicted by DeepPurpose ranged from 5.17 to 7.64, as shown in Table S1. Known targets included CB1, CB2, GPR3, GPR55, GPR6, TRPV1, and PPARs. CBD has demonstrated interaction with CB1 and CB2 receptors when CBD is at doses equivalent to or lower than 1 μm, despite low affinities [30] and has been revealed for its interactions with other targets through experimental studies. While it may be difficult to assert that these binding affinities surpass the known CBD targets, these were in line with, or at least closely approximated, those of known targets.

These targets had ‘Alzheimer’s disease’ phenotype data in the Online Mendelian Inheritance in Man (OMIM) [31], highlighting their strong connection to AD. Furthermore, we detailed the biological activities of these targets and their relevance to AD, with APOE, APP, ADAM10, and PSEN1 being implicated in the signaling pathways of AD according to the Kyoto Encyclopedia of Genes and Genomes (KEGG) [32]. Genetic studies have pointed towards the association of these four genes with familial early-onset AD. Additionally, we found that MPO response to oxidative stress promotes Aβ deposition, tau hyperphosphorylation, and the subsequent loss of synapses and neurons [33], and ENOS has negative regulation of blood pressure, which is associated with AD [34].

#### 2.1.1. Molecular Docking Analysis for CBD-Protein Complex

Before starting the docking analysis, we conducted a re-docking procedure for the known compound on its targets to validate the binding site. The results of this re-docking validation are illustrated in Figure S2 and Table S3, and docking parameters are provided in Table S2. We calculated the docking score between CBD and identified potential targets through Qvina-W [35]. A recent study showed that a docking score of less than −5 kcal/mol indicates a good binding activity between molecule and target, and a docking score of less than −7 kcal/mol indicates a more robust binding activity [36]. Table 2 demonstrated docking scores between CBD and identified targets, and docking scores are observed to be in the range of −8.0 to −6.4. It showed that CBD had the potential to bind all identified targets since all docking scores are less than −5 kcal/mol.

**Figure 1:**
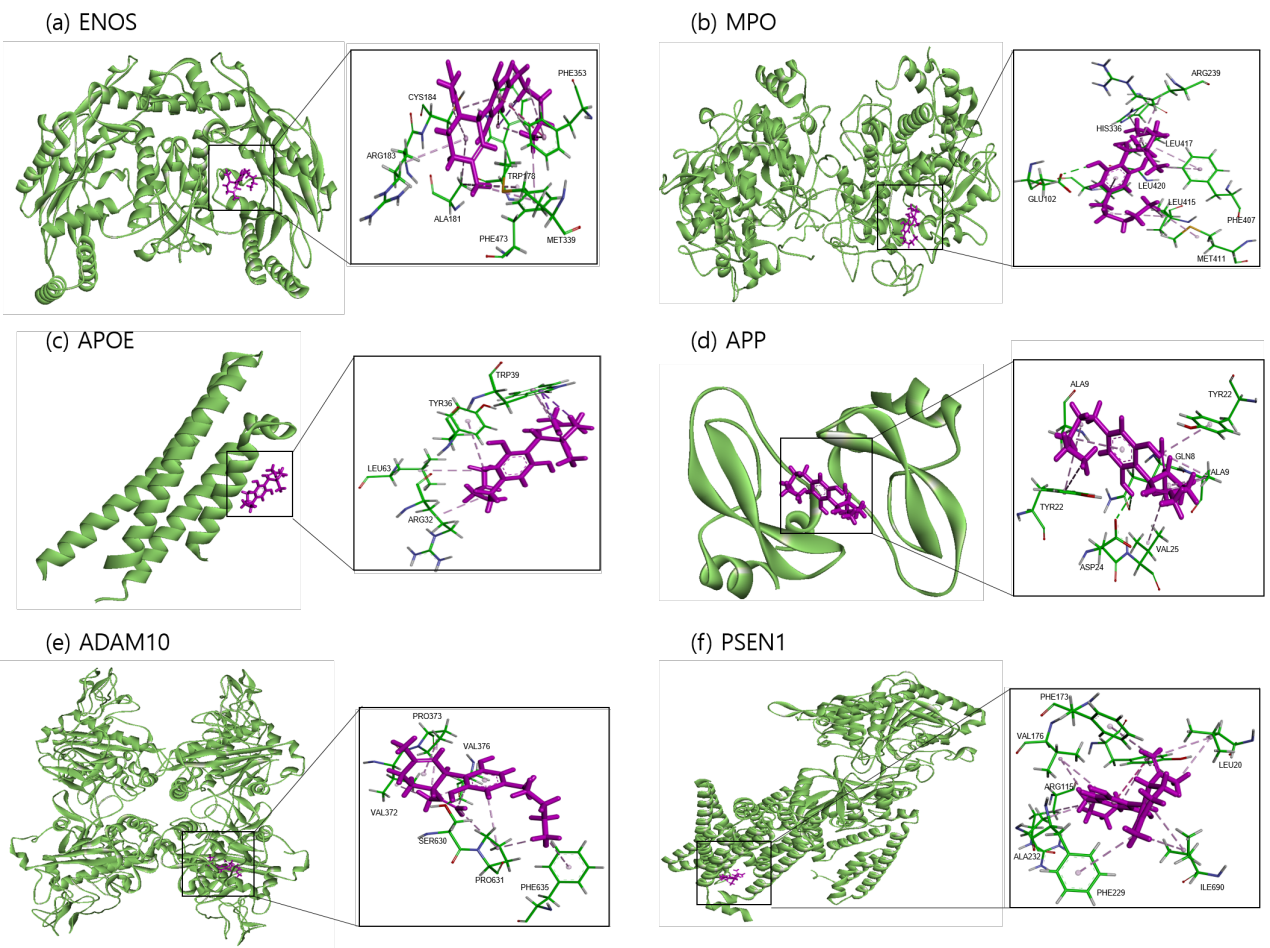
Molecular docking of identified potential targets to CBD: (**a**) ENOS, (**b**) MPO, (**c**) APOE, (**d**) APP, (**e**) ADAM10, and (**f**) PSEN1. CBD is colored magenta, and protein is colored green.

**Figure 2:**
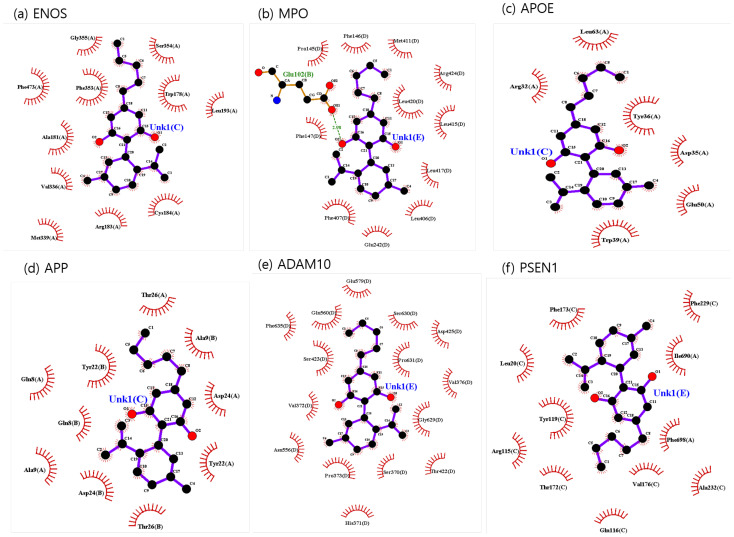
CBD-identified potential targets’ interaction plots: (**a**) ENOS, (**b**) MPO, (**c**) APOE, (**d**) APP, (**e**) ADAM10, and (**f**) PSEN1.

The binding sites involving CBD and each of the identified potential targets were shown in Figure 1 and Figure 2, with further details provided in Table 3. The hydrogen bond and the hydrophobic interaction were important contributors to drug–protein interaction. They associated the binding affinity between ligand–protein interfaces and drug efficacy [37]. The biological activity of the drug lead is proportional to the number of hydrophobic atoms in the active core of the ligand–protein interface [38]. As illustrated in Figure 2 and summarized in Table 3, the number of stabilized hydrophobic interactions in the complexes was as follows: 11 for CBD in complex with ENOS, 11 for MPO, 10 for APOE, 6 for APP, 15 for ADAM10, and 11 for PSEN. CBD-MPO complex was stabilized by a hydrogen bond with 1 residue measured 2.9 Å.

#### 2.1.2. Molecular Dynamics Analysis for CBD-Protein Complex

We used MD simulations to explore various aspects of the ligand–protein complexes, including their internal motion, conformational changes, interaction mechanisms, and binding stability [39]. To determine the stability of the CBD-protein complex, we calculated root-mean-square deviation (RMSD), solvent-accessible surface area (SASA), radius of gyration (Rg), and root-mean-square fluctuation (RMSF) during a 100 ns simulation.

**Figure 3:**
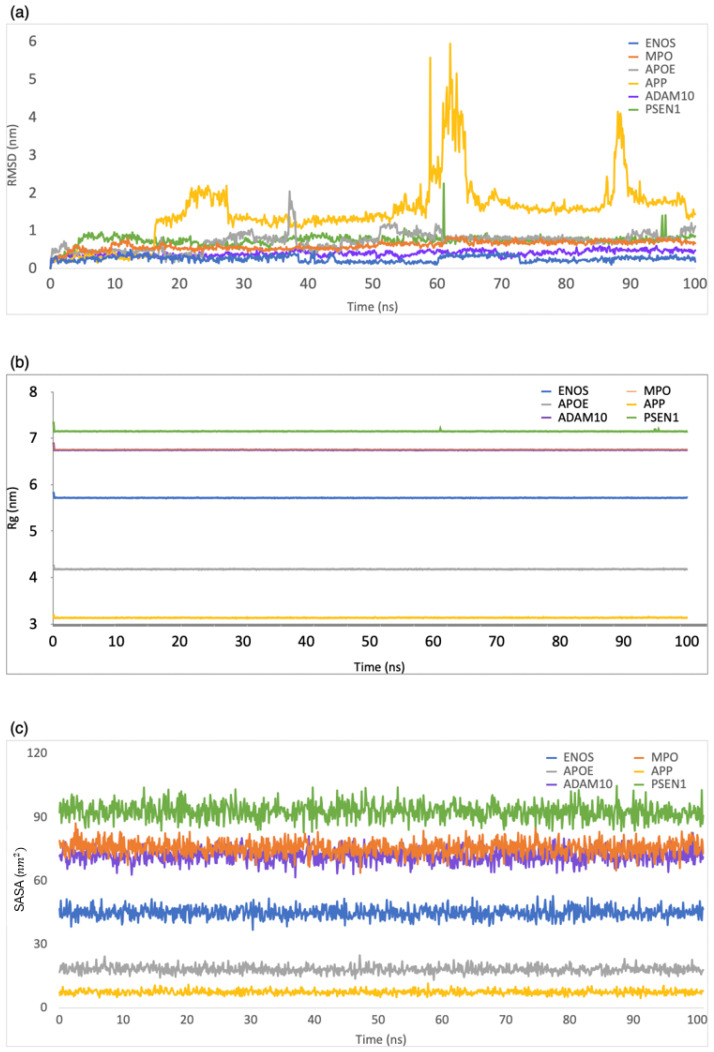
MD simulation analysis of the system of CBD-target complexes over 100 ns: (**a**) RMSD; (**b**) Rg; (**c**) SASA.

RMSD was employed to evaluate the structural stability and deviation of the ligand–protein complexes. As shown in Figure 3a, the range of RMSD values was 0–0.5 nm, 0–0.8 nm, 0–2 nm, 0–5.9 nm, 0–0.6 nm, and 0–2.2 nm for CBD in complex with ENOS, MPO, AOPE, APP, ADAM10, and PSEN, respectively. The analysis of these RMSD values indicated that most complexes remained stable during the simulation period, with RMSD values consistently below 1 nm. This stability showed that the ligand–protein interactions in these complexes were relatively well maintained, with minimal deviation from their initial conformations.

Rg was employed to evaluate the compactness of ligand–protein complexes. As shown in Figure 3b, the range of Rg values was 5.7–5.9 nm, 6.7–6.9 nm, 4.1–4.3 nm, 3.1–3.3 nm, 6.7–6.9 nm, and 7.1–7.3 nm for CBD in complex with ENOS, MPO, AOPE, APP, ADAM10, and PSEN, respectively. The analysis of Rg values indicated that all complexes displayed a low degree of fluctuation, which means a high degree of compactness and stability in the ligand–protein interactions.

The SASA was employed to evaluate the exposure of the ligand–protein complex to the solvent. As shown in Figure 3c, the range of SASA values was 36.8–52.8 nm^2^, 67.7–87.2 nm^2^, 13.2–24.9 nm^2^, 4.6–11.6 nm^2^, 61.6–82.6 nm^2^, and 82.8–104.8 nm^2^ for CBD in complex with ENOS, MPO, AOPE, APP, ADAM10, and PSEN, respectively. The analysis of SASA values indicated the overall stability of all complexes during the simulation period, with only minor fluctuations in SASA values.

**Figure 4:**
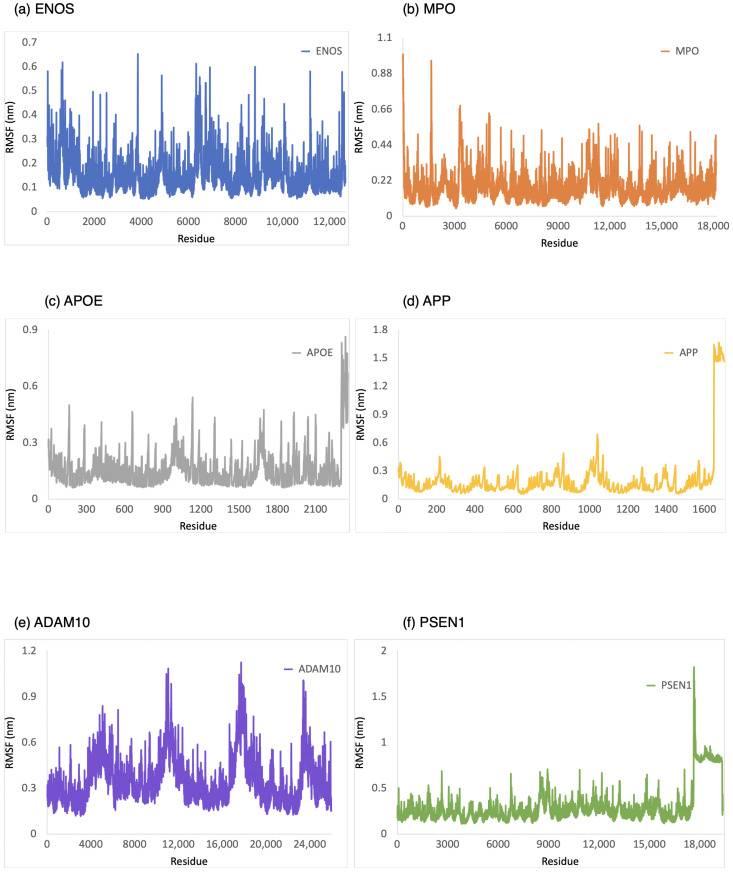
RMSF analysis of the system of CBD-target complexes over 100 ns: (**a**) ENOS; (**b**) MPO; (**c**) APOE; (**d**) APP; (**e**) ADAM10; and (**f**) PSEN1.

The RMSF was employed to provide a conformational dynamics and flexibility of a protein as a function of residue number. We found which residues in the protein exhibit significant fluctuations in their positions when bound to CBD by analyzing the RMSF values. As shown in Figure 4, the range of RMSF values was 0–0.6 nm, 0–1.0 nm, 0–0.8 nm, 0–1.6 nm, 0.1–1.1 nm, and 0.1–1.8 nm for CBD in complex with ENOS, MPO, AOPE, APP, ADAM10, and PSEN, respectively. The RMSF values for these complexes were consistently within the range of 1.8 nm or below. The analysis of RMSF values indicated that these complexes did not undergo significant structural deviations during the simulation, demonstrating good stability.

### 2.2. Generation of CBD Analogs for Each Identified Potential Target

**Figure 5:**
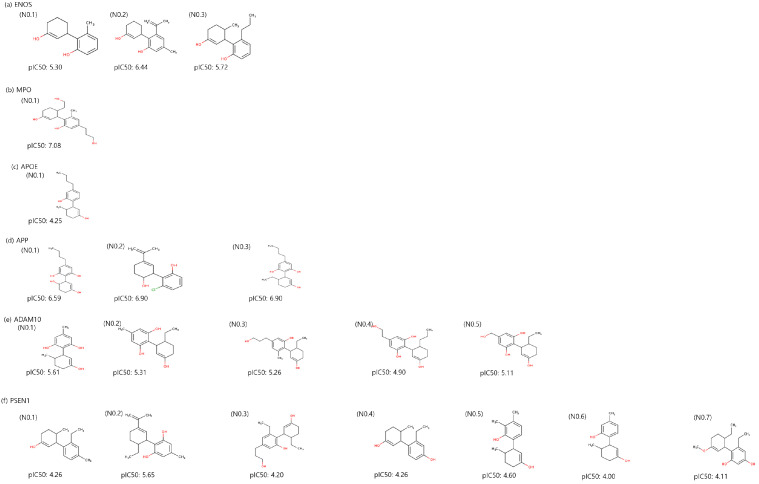
Two-dimensional structure and binding affinity of generated CBD analogs for each identified potential target.

Utilizing a molecular-constrained optimization method, we systematically generated CBD analogs for each identified potential target with a dual focus: minimizing docking scores while optimizing drug-likeness properties and adhering to physicochemical property filters. As a result, we generated three CBD analogs for ENOS and APP, one CBD analog for MPO and APOE, five CBD analogs for ADAM10, and seven CBD analogs for PSEN1, as shown in Figure 5. The $pIC_{50}$ value of CBD analogs, calculated by DeepPurpose, increased overall compared to CBD. The No.2 molecule for ENOS, the No.1 molecule for MPO, the No.2 and 3 molecules for APP, and the No.1 molecule for ADAM10 had the highest $pIC_{50}$ values, and $pIC_{50}$ values are increased by 1.02, 0.80, 1.04, and 0.8 compared to CBD, respectively.

#### 2.2.1. Optimization of Molecular Properties

The molecular-constrained optimization method generated CBD analogs that conform to desired drug-likeness properties and physicochemical property filters. The generated CBD analogs satisfied criteria such as a quantitative estimate of drug-likeness (QED) ≥ 0.5, synthetic accessibility (SAScore) ≤ 6.0, logP ≤ 5.0, and physicochemical property filters, such as pan-assay interference compounds (PAINS) filter [40], BRENK filter [41], NIH filter [42], and ZINC filter. The QED cutoff was based on a study in which oral drugs approved by the FDA have an average of 0.539 QED [43]. The SAScore cutoff was based on a study in which synthesis is difficult if SAS > 6.0 [44]. The cutoff for logP was based on a study in which 90% of oral drugs that have achieved phase II clinical status had logP < 5.0 [45]. We optimized penalized logP (plogP) [46], which was used to prevent abnormally many ring structures and was calculated as follows:

(1) plogP=logP−SAScore−RingPenalty

Additionally, we measured the blood–brain barrier (BBB) permeant of generated CBD analogs using SwissADME [47] due to our target disease being AD. Most of the CBD analogs exhibited a BBB permeant, except for the No.1 molecule for MPO and the No.4 and No.5 molecules for ADAM10.

Table 4 outlines the optimized drug-likeness properties, physicochemical property filters, and BBB permeability of the CBD analogs for each identified potential target. These results collectively showed the performance of the molecular-constrained optimization method in yielding CBD analogs that satisfied the desired properties while retaining the potential for BBB permeation, and it explained the possibility of the drug for AD.

#### 2.2.2. Molecular Docking Analysis for CBD Analog–Protein Complex

CBD analogs had lower or similar docking scores than CBD. Table 5 demonstrated docking scores between CBD analogs and each identified a potential target, and docking scores are observed to be in the range of −8.8 to −6.1. Notably, multiple analogs demonstrated superior docking scores compared to CBD, with three analogs for ENOS, two for ADAM10, and one for MPO and APOE. This result demonstrated the potential of the molecular-constrained optimization method in generating CBD analogs that exhibit enhanced interactions with the targets.

Additionally, given the recognized significance of the endocannabinoid system in AD [48,49], we employed the CIRCE [50] to validate the interaction between CBD analogs and this system. This software predicts whether a query compound acts as a ligand in the endocannabinoid system by comparing its similarity to known ligands interacting with CB1, CB2, and GPRs. The probability prediction of the endocannabinoid system class is expressed as a value ranging from 0 to 1. As shown in Table 5, all CBD analogs exhibited the potential to interact with the endocannabinoid system, having a score of 0.2 or higher. Key substructures of CBD analogs for CB1 and CB2 interactions are illustrated in Figure S1. This result demonstrated that CBD analogs exhibited enhanced interactions with the targets and exhibited interactions with the endocannabinoid system.

**Figure 6:**
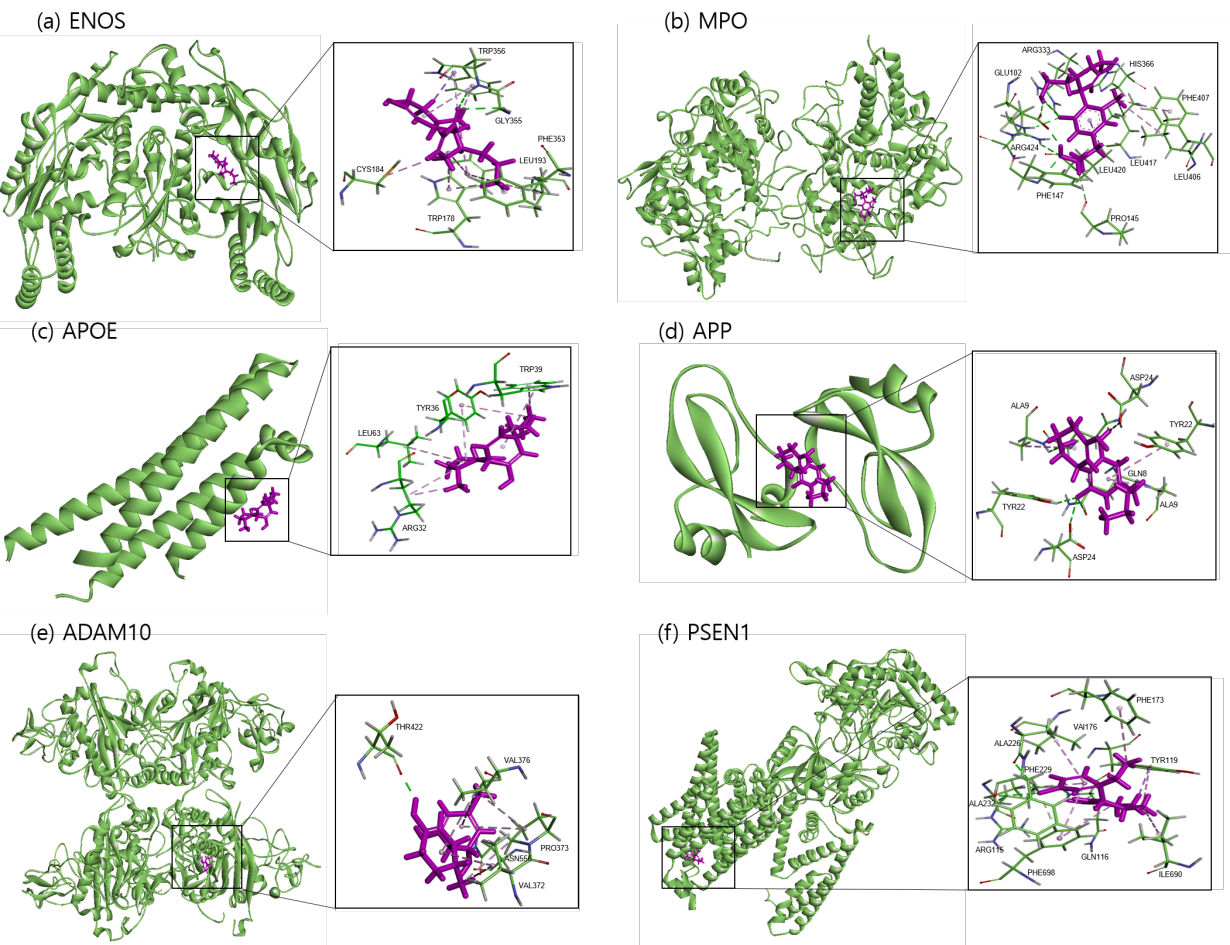
Molecular docking of identified potential targets to CBD analog: (**a**) ENOS, (**b**) MPO, (**c**) APOE, (**d**) APP, (**e**) ADAM10, and (**f**) PSEN1. CBD analog is colored magenta and protein is colored green.

**Figure 7:**
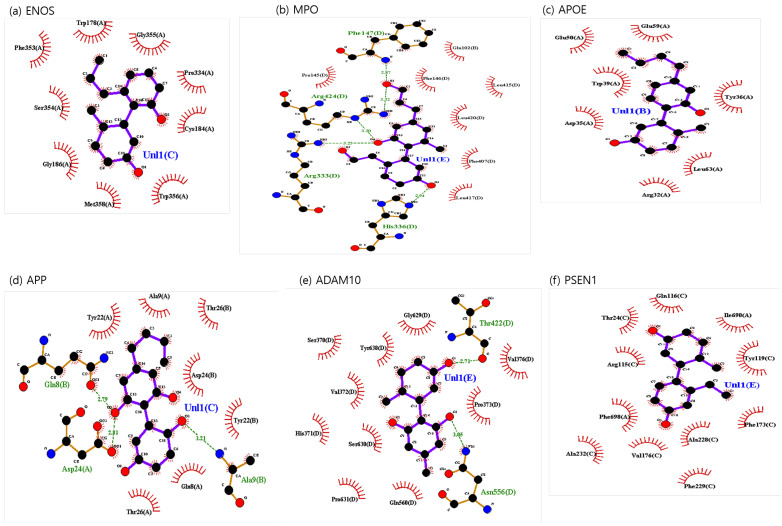
CBD analog-identified potential targets’ interaction plots: (**a**) ENOS, (**b**) MPO, (**c**) APOE, (**d**) APP, (**e**) ADAM10, and (**f**) PSEN1.

We used the No.1 CBD analog in each target for visualization and MD simulation. The binding sites involving the CBD analog and each of the identified potential targets are shown in Figure 6 and Figure 7, with further details provided in Table 6. As shown in Figure 7 and summarized in Table 6, the number of stabilized hydrophobic interactions in the complexes was as follows: 9 for CBD analog in complex with ENOS, 7 for MPO, 7 for APOE, 7 for APP, 10 for ADAM10, and 11 for PSEN. The number of stabilized hydrogen bonds in the complexes was as follows: four for CBD analog in complex with MPO, three for APP, and two for ADAM10.

The structural analysis revealed distinct interactions between CBD analogs and the identified targets compared to the CBD complex. The CBD analog-ENOS complex shared the same four residues as the CBD complex: Gly355, Cys184, Ser354, and Phe353. CBD analog-MPO complex shared the same six residues as the CBD complex: Leu415, Phe407, Leu417, Pro145, Phe147, and Arg424. In these residues, Phe147 and Arg424 changed hydrophobic interaction to the hydrogen bond. The CBD analog-APOE complex shared all residues identically with the CBD complex. The CBD analog-APP complex shared all residues identically with the CBD complex. In these residues, Ala9(B), Asp24(A), and Gln8(B) shifted hydrophobic interaction to the hydrogen bonds. The CBD analog-ADAM10 complex shared the same nine residues: Thr422, Pro373, Asn556, Gln560, Pro631, Ser630, His371, Val372, and Gly629. In these residues, Thr422 and Asn556 shifted hydrophobic interaction to the hydrogen bond. The CBD analog-PSEN1 complex shared the same nine residues: Gln116, Ile690, Tyr119, Phe173, Phe229, Val176, Ala232, Phe698, and Arg115. Overall, this result demonstrated the potential of CBD analogs to foster distinct binding interactions, potentially influencing the affinity of the compounds for these targets.

#### 2.2.3. Molecular Dynamics Analysis for CBD Analog–Target Complex

**Figure 8:**
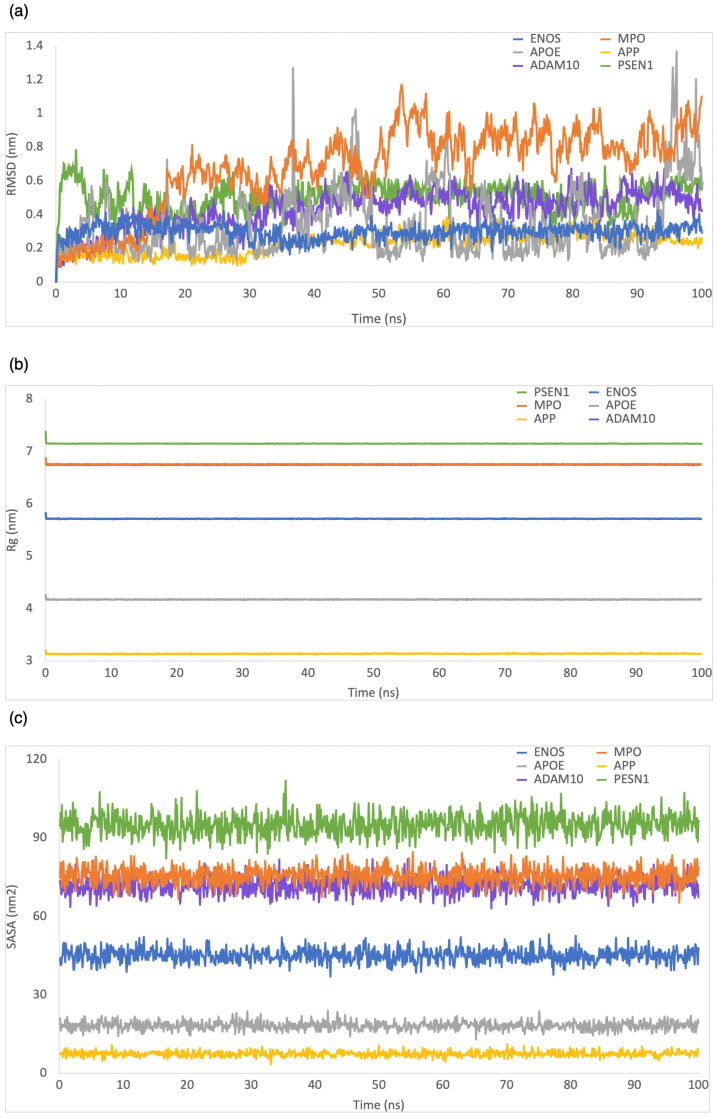
MD simulation analysis of the system of CBD analog–target complexes over 100 ns: (**a**) RMSD; (**b**) Rg; (**c**) SASA.

**Figure 9:**
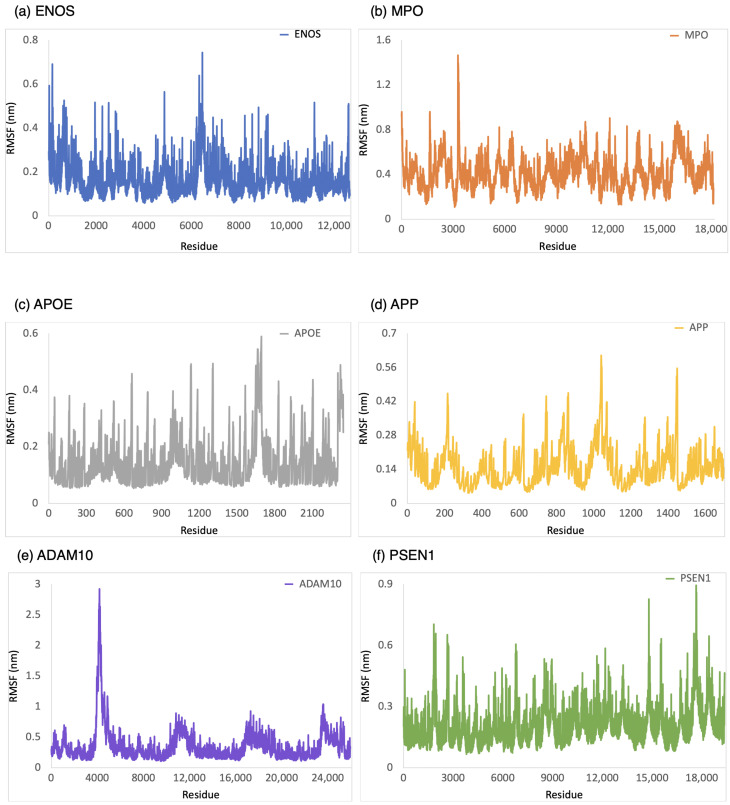
RMSF analysis of the system of CBD analog–target complexes over 100 ns: (**a**) ENOS, (**b**) MPO, (**c**) APOE, (**d**) APP, (**e**) ADAM10, and (**f**) PSEN1.

We measured the binding stability of the CBD analog–protein complex using MD simulation during a 100 ns simulation. As shown in Figure 8, the range of RMSD values was 0–0.4 nm, 0–1.2 nm, 0–1.4 nm, 0–0.4 nm, 0–0.7 nm, and 0–0.8 nm for the CBD analog in complex with ENOS, MPO, AOPE, APP, ADAM10, and PSEN, respectively. The range of Rg values was 5.7–5.9 nm, 6.7–6.9 nm, 4.1–4.3 nm, 3.1–3.3 nm, 6.7–6.8 nm, and 7.1–7.4 nm for the CBD analog in complex with ENOS, MPO, AOPE, APP, ADAM10, and PSEN, respectively. The range of SASA values was 36.8–53.1 nm^2^, 65.2–84.6 nm^2^, 12.9–24 nm^2^, 3.4–11 nm^2^, 63–81.9 nm^2^, and 82.1–111.9 nm^2^ for the CBD analog in complex with ENOS, MPO, AOPE, APP, ADAM10, and PSEN, respectively. As shown in Figure 9, the range of RMSF values was 0–0.7 nm, 0.1–1.4 nm, 0–0.6 nm, 0–0.6 nm, 0.1–2.9 nm, and 0–0.8 nm for the CBD analog in complex with ENOS, MPO, AOPE, APP, ADAM10, and PSEN, respectively.

Similar to the CBD-target complexes, the majority of CBD analog–target complexes showed good stability during MD simulations, as evidenced by RMSD values consistently below 1 nm, low degrees of fluctuation in Rg and SASA, and RMSF values generally within the range of 3 nm or lower. However, there were some notable differences compared to the CBD-target complexes. In the case of the CBD analog-ADAM10 complex, the RMSF value was higher than the CBD complex, indicating that this complex exhibited slightly more flexibility in specific residues. The RMSF values of the CBD complex with APOE, APP, and PSEN1 showed a sharp change at the end of the residue, which may indicate instability. In contrast, the CBD analog with these targets exhibited stability, indicating that the analogs might have improved the binding interactions compared to CBD. The CBD analog-APP complex demonstrated better stability when compared to the CBD-APP complex.

## 3. Discussion

This study found that CBD and CBD analogs are promising in AD treatment. Our analysis showed potential protein targets associated with AD, and the computational generation of CBD analogs has shown potential for enhancing the stability and binding interactions with these potential targets. However, we noted a few limitations that need to be considered. First, we acknowledged the variability in complex stability, as indicated by high RMSF values at the end of binding site residues for three CBD-target complexes and one CBD analog–target complex. These variations might indicate potential challenges in achieving consistent binding stability with all targets, and suggest that future MD simulations might benefit from longer durations than the commonly employed 100 ns to capture more comprehensive insights into stability. Additionally, we recognized that our binding affinity calculations depended on the DeepPurpose model, which may not be the optimal tool for precisely measuring binding affinity. Further refinement in binding affinity prediction models could enhance the accuracy of our findings.

Nevertheless, these findings underscore the potential for CBD to play a more expansive role in the development of AD treatments and excited possibilities for CBD analogs as drug candidates to improve binding interactions and stability with AD-associated protein targets through various analyses. These results will require further validation through experimental investigations to ascertain their real-world therapeutic applications and safety profiles. While CBD analogs have shown promise by satisfying critical drug-likeness properties, additional considerations should be given to optimizing ADME properties and bioavailability. In future steps, conducting in vitro and in vivo experiments to validate the observed effects of CBD and CBD analogs on the identified targets is essential. Moreover, exploring potential therapeutic effects with other AD treatments and investigating long-term effects are avenues for further research. Therefore, while this study has provided valuable insights into the potential of CBD and CBD analogs for AD treatment, it represents just the beginning. Addressing the challenges in optimizing ADME properties and bioavailability and experimental validation in vitro and in vivo are essential to translating these findings into practical and effective AD treatments.

## 4. Materials and Methods

### 4.1. Potential CBD Target Identification Related to AD

#### 4.1.1. Dataset

We used the drug–target interaction and disease–target association datasets to identify CBD targets. Our drug–target interaction dataset was built by integrating DrugBank [51] and Therapeutic Targets Database (TTD) [52]. We only selected pairs with representation, such as SMILES [53] of drugs and the sequence of targets, to input the drug–target binding affinity prediction model. As a result, our drug–target interaction dataset was composed of 18,634 drugs and 3435 targets.

We used OMIM as the disease–target association dataset. OMIM has a relationship between genes and phenotypes, which are information on diseases and symptoms caused by these genes. We mapped the OMIM ID of genotype data with the UniProt ID [54] of target data through HUGO Gene Nomenclature Committee (HGNC) [55] because target names in our drug–target interaction dataset were represented by UniProt ID. As a result, our disease–target association dataset was composed of 7775 genotype data and 16,619 phenotype data.

#### 4.1.2. Drug–Target Binding Affinity Prediction Method

The drug–target binding affinity prediction method was based on the principle that drugs with similar molecular structures tend to bind to targets with similar functions and structures. The workflow for identifying potential CBD targets associated with AD consisted of three steps. First, we extracted similar molecules of CBD in our drug–target interaction dataset. Molecular similarity to CBD was calculated using the average of fingerprint Tanimoto similarities computed with Morgan and MACCS fingerprints via RDKit [56]. Second, we quantitatively measured the binding potential between CBD and BD-like molecules’ targets in our drug–target interaction dataset using DeepPurpose (MPNN [57]-CNN [58] pre-trained model). Third, we filtered candidate targets based on binding affinity, calculated on DeepPurpose, and searched targets related to AD among candidate targets in our disease–target association dataset.

### 4.2. CBD Analog Generation Using Our Molecular-Constrained Optimization Method

**Figure 10:**
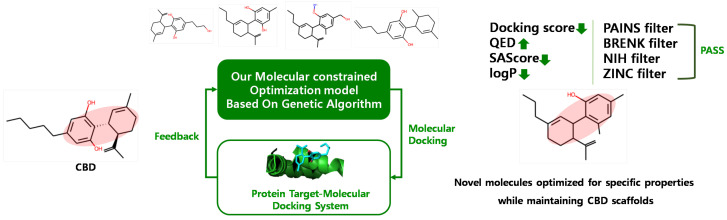
Workflow for generating CBD analogs for each target.

We generated CBD analogs that optimized each identified potential target found by our target identification method. The workflow for generating CBD analogs was shown in Figure 10. It consisted of two steps. First, we generated novel molecules, called CBD analogs, which preserved the Murcko scaffold of CBD [59] and optimized multiple drug-likeness properties, such as the QED, SAScore, plogP, and logP, using our molecular-constrained optimization method. In this work, we additionally optimized physicochemical property filters, such as PAINS, BRENK, NIH, and ZINC filters, to prevent the generation of toxic and synthetically infeasible subgroups in small molecules [60]. Second, we calculated the docking score between generated CBD analogs and the target, and then gave the docking score as feedback from the first step.

### 4.3. The Results Analysis Method

#### Molecular Docking

To simulate molecular docking, we prepared small molecules and target proteins using OpenBabel [61] and Biovia discovery studio visualizer (version 21.20298). The OpenBabel had converted SMILES of molecules to 3D structures. The Pymol software (version 2.5.7) removed ligand residues from proteins. To delineate the binding sites of our identified targets, we employed PrankWeb [62] to predict potential pockets within these target proteins. Subsequently, we retrieved ligand structures of known inhibitors for these targets from the Protein Data Bank (PDB) website (https://www.rcsb.org/ (accessed on 23 July 2023)) and referenced previous docking papers. We specifically defined the binding sites from the multiple pockets generated by PrankWeb by considering those containing binding residues highlighted in the literature or known inhibitors cataloged in the PDB. We used the Qvina-W to reveal the interaction between active small molecules and target proteins. After docking simulation, we visualized the small molecules–protein complex to Biovia discovery studio visualizer and the interaction site between molecules and ligands to LIGPLOT (version 2.2) [63].

### 4.4. Molecular Dynamics Simulation

The MD simulation described the conformational changes and structural safety of the ligand–protein complex during the binding process. Moreover, they further provided evidence for the binding mode of small molecules and receptor proteins [64]. We used CBD and each CBD analog, the top-predicted docking pose with the best docking score among ten times docking simulations, as the starting point for an MD simulation. We generated the initial protein complex structure using CHARMM-GUI [65] and performed MD simulation using GROMACS (version 22.4) [66]. MD simulations were performed with periodic boundary conditions. The van der Waals forces and Lennard-Jones interactions involved a cut-off distance of 1.2 Å. The first stage optimized each system structure using 5000 iterations (5 ps) with the steepest descent algorithm. After minimizing the energy, the system equilibrated the NVT (constant particle number, volume, and temperature) and the NPT (constant particle number, pressure, and temperature). NVT and NPT were ensembled for 125 ps. Temperature coupling was set to 1ps and pressure coupling to 5 ps. Finally, the MD simulation was run for 100 ns under constant pressure and temperature. We analyzed RMSD, Rg, SASA, and RMSF for complex structures obtained from the 100 ns MD simulations using GROMACS (version 22.4).

## Figures and Tables

**Table 1:** Identified potential targets for CBD associated with AD.

Uniprot ID	Target Name	Binding Affinity (${\mathrm{pIC}}_{50}$)
P29474	ENOS	5.42
P05164	MPO	7.00
P02649	APOE	5.85
P05067	APP	5.86
O14672	ADAM10	4.81
P49768	PSEN1	6.78

**Table 2:** CBD docking score (kcal/mol) to identify potential targets related to AD by molecular docking.

PDB ID	Target Name	Docking Score (kcal/mol)
3NOS	ENOS	−8.0
1DNW	MPO	−6.6
1B68	APOE	−5.8
1AAP	APP	−6.4
6BE6	ADAM10	−7.0
5A63	PSEN1	−7.9

**Table 3:** Molecular interaction of identified target active site with CBD.

Complex Name	Residues of Hydrophobic Interaction	Residues of ydrogen Bond (Å)
CBD-ENOS	Gly355, Ser354, Phe473, Phe353, Trp178, Leu193, Ala181, Val336, Mel339, Arg183, Cys184	-
CBD-MPO	Pro145, Phe146, Met411, Arg424, Leu420, Leu415, Phe147, Leu417, Leu406, Phe407, Glu242	Glu102 (2.9 Å)
CBD-APOE	Leu63, Arg32, Tyr36, Asp35, Glu50, Trp39	-
CBD-APP	Thr26 (A), Ala9 (A), Tyr22 (A), Gln8 (A), Asp24 (A), Gln8 (B), Ala9 (B), Tyr22 (B), Asp24 (B), Thr26 (B)	-
CBD-ADAM10	Glu579, Gln560, Phe635, Ser630, Asp425, Pro631, Ser423, Val376, Gly629, Val372, Asn556, Pro373, Ser350, Thr422, His371	-
CBD-PSEN1	Phe229, Phe173, Ile690, Leu20, Tyr119, Arg115, Phe698, Thr172, Val176, Ala232, Gln116	-

**Table 4:** Optimized molecular properties, filters, and BBB permeant of CBD analogs for each identified potential target.

Target Name	No.	QED	SAScore	plogP	logP	Filters (PAINS, BRENK, NIH, ZINC)	BBB Permeant
ENOS	No.1	0.73	3.28	0.12	3.41	Pass	Yes
	No.2	0.80	3.58	0.86	4.44	Pass	Yes
	No.3	0.83	3.65	0.64	4.30	Pass	Yes
MPO	No.1	0.65	3.80	−0.86	2.94	Pass	No
APOE	No.1	0.82	3.50	1.18	4.69	Pass	Yes
APP	No.1	0.68	3.69	−0.57	3.12	Pass	No
	No.2	0.85	3.80	−0.02	3.78	Pass	Yes
	No.3	0.73	3.67	1.11	4.78	Pass	Yes
ADAM10	No.1	0.69	3.77	−0.41	3.36	Pass	Yes
	No.2	0.74	3.76	−0.01	3.75	Pass	Yes
	No.3	0.76	3.73	0.23	3.97	Pass	Yes
	No.4	0.67	3.82	−0.45	3.36	Pass	No
	No.5	0.67	3.82	−0.89	2.93	Pass	No
PSEN1	No.1	0.79	3.58	0.92	4.51	Pass	Yes
	No.2	0.82	3.85	0.95	4.81	Pass	Yes
	No.3	0.73	3.79	0.43	4.22	Pass	Yes
	No.4	0.81	3.72	0.18	3.91	Pass	Yes
	No.5	0.76	3.73	0.22	3.96	Pass	Yes
	No.6	0.75	3.60	0.05	3.65	Pass	Yes
	No.7	0.87	3.79	0.29	0.40	Pass	Yes

**Table 5:** Docking score of CBD analogs for each identified potential target. Potential1 is the potential of CBD analog to interact with a CB1 or CB2. Potential2 is the potential of CBD analog preferentially binds to cannabinoid receptors compared to other GPRs.

Target Name	No.	Docking Score (kcal/mol)	Potential1	Potential2
ENOS	No.1	−8.8	0.33	0.4
	No.2	−8.7	0.3	0.68
	No.3	−8.2	0.3	0.46
MPO	No.1	−7.4	0.32	0.54
APOE	No.1	−6.1	0.61	0.82
APP	No.1	−6.5	0.63	0.83
	No.2	−6.1	0.21	0.44
	No.3	−6.1	0.63	0.83
ADAM10	No.1	−7.0	0.35	0.54
	No.2	−7.6	0.37	0.57
	No.3	−7.2	0.32	0.54
	No.4	−6.9	0.34	0.62
	No.5	−6.7	0.36	0.59
PSEN1	No.1	−8.0	0.22	0.48
	No.2	−7.9	0.42	0.77
	No.3	−7.9	0.32	0.54
	No.4	−7.8	0.23	0.43
	No.5	−7.7	0.43	0.56
	No.6	−7.7	0.32	0.47
	No.7	−7.5	0.33	0.53

**Table 6:** Molecular interaction of identified target active site with CBD analogs.

Complex Name	Residues of Hydrophobic Interaction	Residues of Hydrogen Bond (Å)
CBD analog	Trp178, Gly355, Pre334,	-
-ENOS	Cys184, Trp356, Mel358,	
	Gly186, Ser354, Phe353	
CBD analog	Glu102, Leu415,	Phe147 (2.57 Å), Arg424 (3.33 Å),
-MPO	Phe146, Leu420, Phe407,	Arg333 (3.25 Å),
	Leu417, Pro145, Phe147	His336 (2.94 Å)
CBD analog	Glu59, Tyr36, Leu63	
-APOE	Arg32, Asp35, Trp39, Glu50	-
CBD analog	Ala9 (A), Thr26 (B), Tyr22,	la9 (B) (3.21 Å)
-APP	Asp24 (B), Ty422, Gln8 (A), Thr26 (A)	Asp24 (A) (2.81 Å), Gln8 (B) (2.79 Å)
CBD analog	Gly629, Try638, Ser370,	
-ADAM10	Val372, Ser630, His371,	Thr422 (2.71 Å), Asn556 (3.05 Å)
	Pro631, Gln560, Pro373, Val376	
CBD analog	Gln116, Ile690, Tyr119,	
-PSEN1	Phe173, Ala228, Phe229, Val176,	-
	Ala232, Phe698, Arg115, Thr24	

## Data Availability

The protein structures of identified targets were acquired online from RCSB Protein Data Bank, including ENOS (PDB ID: 3NOS), MPO (PDB ID: 1DNW), APOE (PDB ID: 1B68), APP (PDB ID: 1AAP), ADAM10 (PDB ID: 6BE6), and PSEN1 (PDB ID: 5A63).

## Supplementary Materials

The supporting information can be downloaded at: https://www.mdpi.com/article/10.3390/ijms242116013/s1. References [67,68,69,70,71] are cited in the supplementary materials.
