# Supplementary material for: An In Silico Study for Expanding the Utility of Cannabidiol in Alzheimer’s Disease Therapeutic Development
Source: Int J Mol Sci. 2023 Nov 6;24(21):16013. doi: 10.3390/ijms242116013 (PMC10648567; doi:10.3390/ijms242116013)
Supplement: Supplementary file 1 [file ijms-24-16013-s001.zip › ijms-2664915-supplementary.pdf]

Supplementary information

An in silico study for expanding the utility of cannabidiol in Alzheimer's disease therapeutic development

Table S1. Binding affinity with known CBD targets and CBD.

Uniprot ID	Target name	Binding affinity (pIC_{50})
Q96F85	CB1	7.64
P34972	CB2	5.17
P46089	GPR3	6.35
Q9Y2T6	GPR55	5.51
P46095	GPR6	6.32
Q8NER1	TRPV1	7.26
Q07869	PPARα	6.08
Q03181	PPARβ	6.49
P37231	PPARγ	6.68

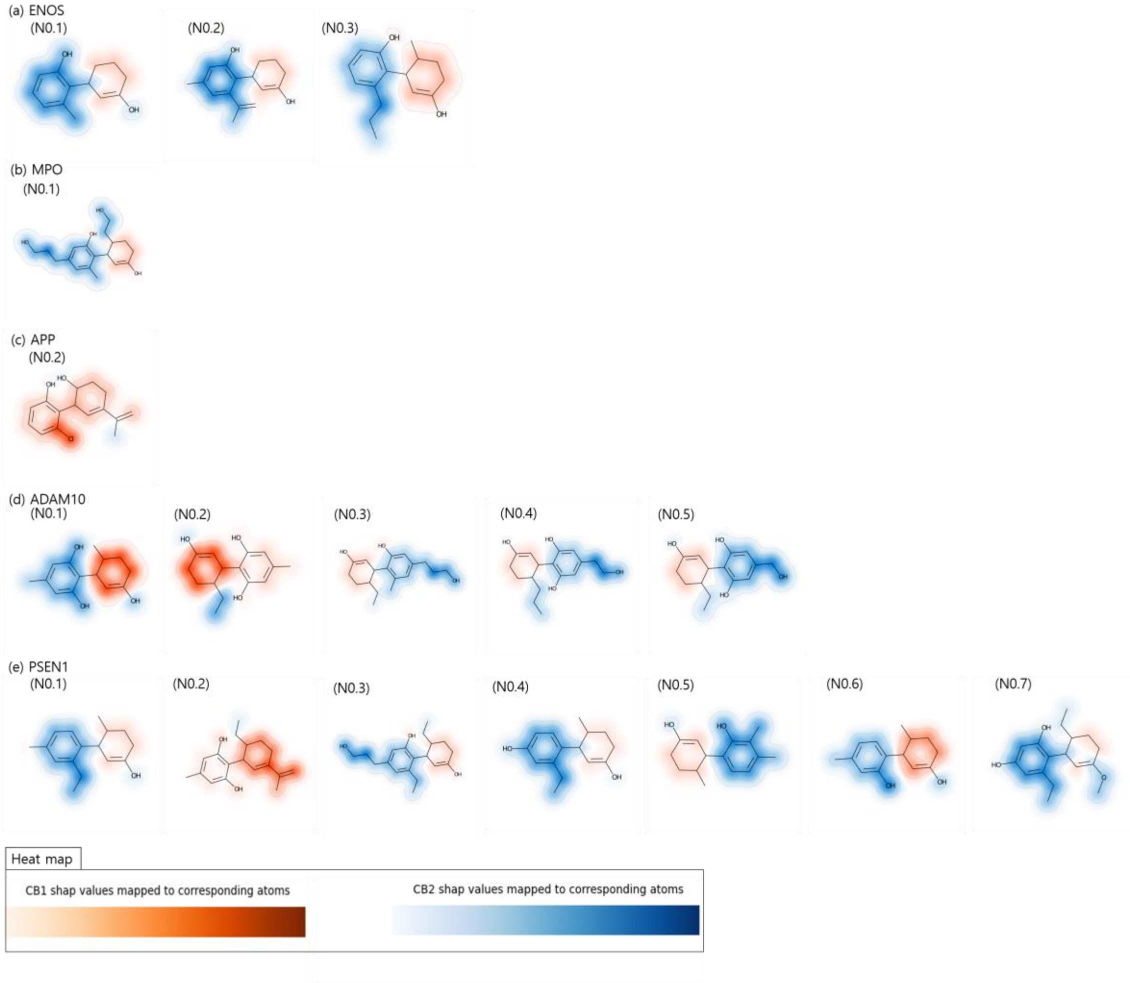

Figure S1. 2D structure of CBD analogs with the SHAP analysis indicating the essential substructures for CB1 and CB2. In the heat map, orange and blue colors indicate significant substructures for interacting CB1 or CB2 ligands, respectively. No.1 molecule for APOE, NO.1 and No.3 molecules for APP are non-selective ligands for CB1 and CB2, so these molecules did not analyze SHAP.

Table S2. Docking parameters for each potential target.

Target name (PDB ID)	Coordinates (x, y, z)	Grid box size (x, y, z)
ENOS (3NOS)	9.962, 11.135, 49.762	58, 70, 88
MPO (1DNW)	28.176, 9.341, 45.441	70, 66, 64
APOE (1B68)	-4.455, 11.313, 21.306	34, 60, 40
APP (1AAP)	14.722, 16.068, 33.687	38, 40, 62
ADAM10 (6BE6)	39.75, 64.801, 18.497	46, 42, 50
PSEN1 (5A63)	126.264, 107.697, 122.218	60, 80, 64

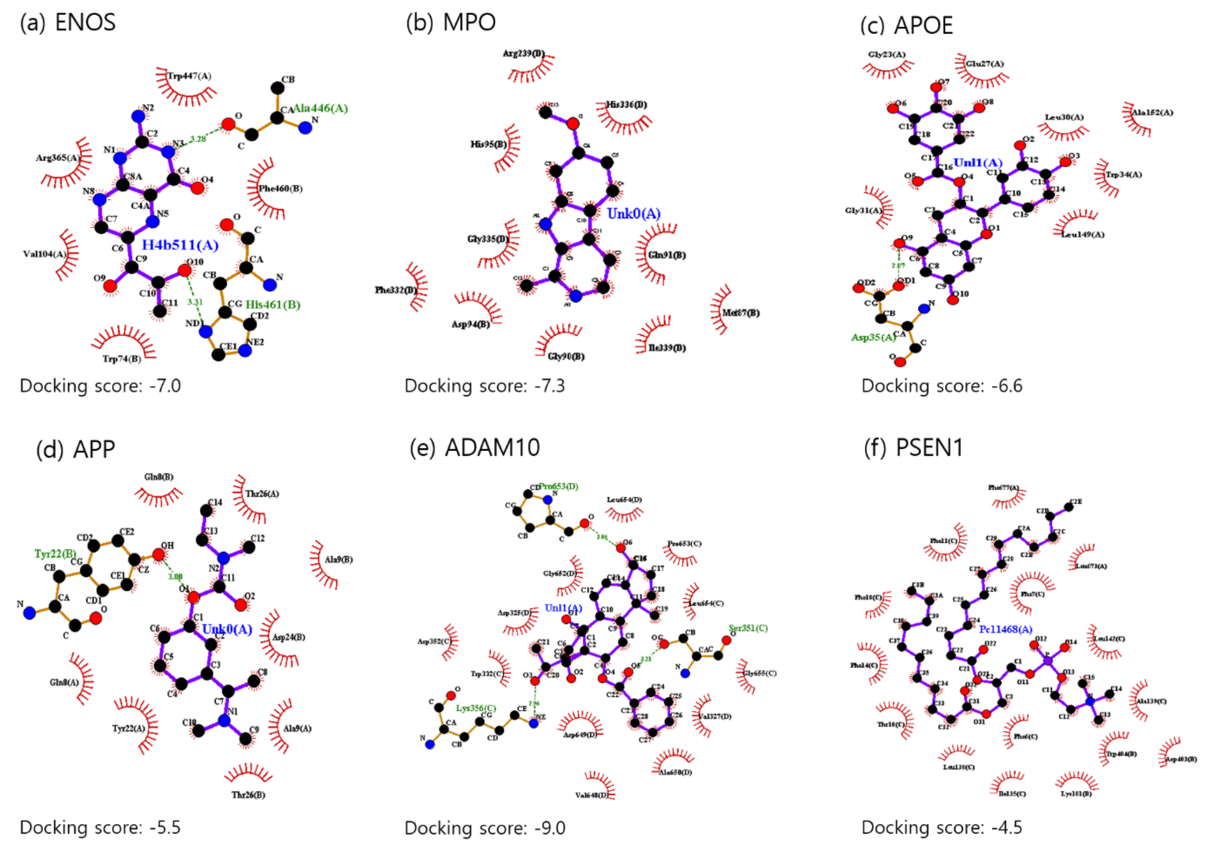

Figure S2. Docking validation. Re-docking of identified potential targets with existing compounds. Existing compound retrieved in PDB and related papers.

Table S3. Comparing between active residues based on source and from re-docking.

Target name (PDB ID)	Compound name	Known active residues based on source	Maintained residues from re-docking	Source
ENOS (3NOS)	Sapropterin	hydrogen bond: Trp447, Ala446<br>hydrophobic interaction: Arg365, Phe460, Ser102, Glu463, Trp445	hydrogen bond: Ala446<br>hydrophobic interaction: Trp447, Arg365, Phe460	PDB
MPO (1DNW)	Harmine	hydrophobic interaction: Gln91, Glu102, His95	hydrophobic interaction: Gln91, His95	[1]
APOE (1B68)	Epicatechin Gallate	MPO's active residue: Trp34, Leu148, Leu149, Trp26, Leu30, Ala152<br>hydrogen bond: Gln156, Asp35<br>hydrophobic interaction: Leu28, Glu27, Trp26, Asp153, Leu159	hydrogen bond: ASP35<br>hydrophobic interactions: Glu27, Ala152, Trp26	[2,3]
APP (1AAP)	rivastigmine	Active residue: Ser6, Glu7, Gln8, Tyr22, Phe23, Asp24, Val25	hydrogen bond: Trp22<br>hydrophobic interactions: Gln8, Tyr22, Asp24	[4]
ADAM10 (6BE6)	Calotropone	hydrogen bond: Asp651<br>hydrophobic interaction: Val333, Leu654(c), Leu654(d), Pro392, His393, Gln439, Ser395, Pro392	hydrophobic interaction: Leu654(c), and Leu654(d)	[5]
PSEN1 (5A63)	1,2-Diacyle-sn-glycero-3-phosphocholine	hydrogen bond: Lys101<br>hydrophobic interaction: Leu142, Phe6, Trp404, Ile408, Val131, Val393, Fhe411, Val94, Ala98, Val97, Ile135, Ala139	hydrophobic interaction: Phe6, Trp404, Ile135, Ala139.	PDB

Reference

[1] Bensalem, Sihem, et al. "Inhibition of myeloperoxidase activity by the alkaloids of Peganum harmala L.(Zygophyllaceae)." Journal of ethnopharmacology 154.2 (2014): 361-369.

[2] Petros, Andrew M., et al. "Fragment-based discovery of an apolipoprotein E4 (apoE4) stabilizer." Journal of Medicinal Chemistry 62.8 (2019): 4120-4130.

[3] Bano, Saddia, et al. "In Silico Identification of Novel Apolipoprotein E4 Inhibitor for Alzheimer's Disease Therapy." Current Computer-Aided Drug Design 15.1 (2019): 97-103.

[4] Rahman, Md Saidur, et al. "In vivo neuropharmacological potential of gomphandra tetrandra (wall.) sleumer and in-silico study against β-amyloid precursor protein." Processes 9.8 (2021): 1449.

[5] Purnama, Agnia, et al. "Molecular docking investigation of calotropone as a potential natural therapeutic agent against pancreatic cancer." Journal of Advanced Pharmaceutical Technology & Research 13.1 (2022): 44.
